# Recent Developments on the Crosstalk Between STAT3 and Inflammation in Heart Function and Disease

**DOI:** 10.3389/fimmu.2018.03029

**Published:** 2018-12-19

**Authors:** Mazen Kurdi, Carlos Zgheib, George W. Booz

**Affiliations:** ^1^Faculty of Sciences, Department of Chemistry and Biochemistry, and The Laboratory of Experimental and Clinical Pharmacology, Lebanese University, Beirut, Lebanon; ^2^Laboratory for Fetal and Regenerative Biology, Department of Surgery, School of Medicine, University of Colorado Denver, Anschutz Medical Campus and Colorado Children's Hospital, Aurora, CO, United States; ^3^Department of Pharmacology and Toxicology, University of Mississippi Medical Center, Jackson, MS, United States

**Keywords:** cardiac remodeling, myocarditis, heart failure, peripartum cardiomyopathy, cytokines, immunity

## Abstract

The transcription factor STAT3 has a protective function in the heart. Until recently, the role of STAT3 in hypertension-induced cardiac hypertrophy was unsettled. Earlier studies revealed that global reduction of STAT3 activity reduced cardiac hypertrophy with hypertension, but caused a disruption of myofilaments and increased contractile dysfunction. However, newer studies with cardiomyocyte-specific deletion of STAT3 indicate that STAT3 does not cause cardiac hypertrophy with increased blood pressure. Rather, cardiac STAT3 is important for maintaining metabolic homeostasis, and loss of STAT3 in cardiomyocytes makes the heart more susceptible to chronic pathological insult, for example by disrupting glucose metabolism and protective signaling networks via the upregulation of certain microRNAs. This scenario has implications for understanding peripartum cardiomyopathy as well. In viral myocarditis, STAT3 opposes the initiation of the dilated phenotype by maintaining membrane integrity via the expression of dystrophin. STAT3 signaling was also found to attenuate myocarditis by polarizing macrophages to a less inflammatory phenotype. On the other hand, STAT3 contributes to immune-mediated myocarditis due to IL-6-induced complement component C3 production in the liver, as well as the differentiation of Th17 cells, which play a role in initiation and development of myocarditis. Besides canonical signaling pathways, unphosphorylated STAT3 (U-STAT3) and redox-activated STAT3 have been shown to couple to transcription in the heart. In addition, tissue signaling cytokines such as IL-22 and IL-17 have been proposed to have actions on the heart that involve STAT3, but are not fully defined. Understanding the novel and often protective aspects of STAT3 in the myocardium could lead to new therapeutic approaches to treat heart disease.

## Introduction

**Figure 1:**
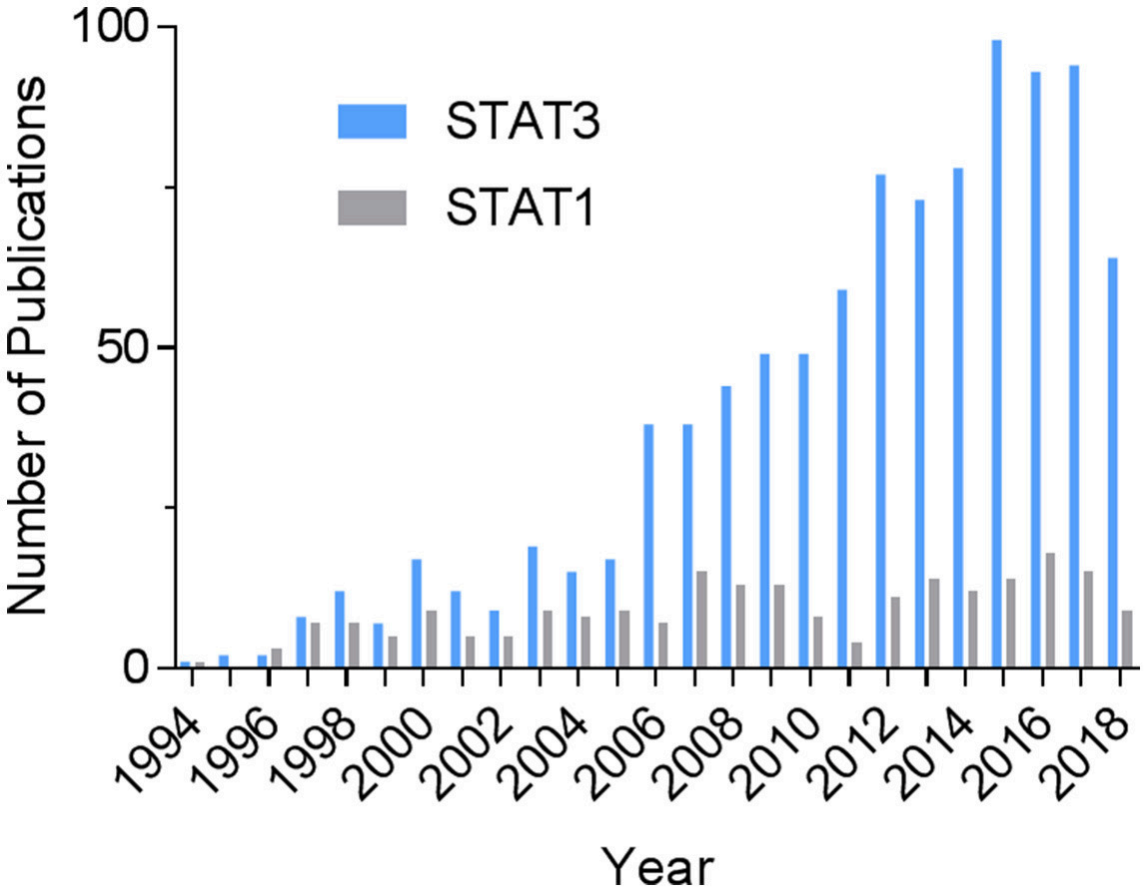
Historical record of STAT3-related publications in the heart. The PubMed databased was searched on August 7, 2018 using the terms “STAT3 and (cardiac or heart)” or, for comparison, “STAT1 and (cardiac or heart).”

The transcription factor signal transducer and activator of transcription 3 (STAT3) continues to excite much interest in heart research. A PubMed search on August 7, 2018 using the terms “STAT3 and (cardiac or heart)” shows a steady increase in the number of publications over time (Figure 1). The total number was more than 4.4 times that of STAT1 and 6.3 times that of STAT5. Activation of STAT3 in the heart has been linked to cardiac protective mechanisms that constitute the various forms of pre-and post-conditioning to protect against ischemia-reperfusion injury (1). Much of the protective actions of STAT3 are attributable to the induction of anti-inflammatory and survival genes. Besides that, STAT3 has been demonstrated to have direct protective effects in mitochondria, which were first demonstrated in cardiomyocytes (2). The reader is directed to several recent reviews that tackle these aspects of STAT3 signaling in the heart, as well as covering the topic of its posttranslational modifications (1–3). In the present review, we focus on recent developments addressing the importance of STAT3 in cardiac hypertrophy, myocardial infarction (MI), heart failure, and peripartum cardiomyopathy, with particular focus on the contribution of immunity and inflammation. Related to this, we provide an update on novel aspects of nuclear STAT3 signaling, namely its redox activation and unphosphorylated (U-STAT3) signaling, as well as the role of STAT3 in the actions of IL-22 and IL-17 on the heart.

## Cardiac Hypertrophy and Heart Failure

In response to increased blood pressure, the heart undergoes cardiac hypertrophy, which is an independent risk factor for morbidity and mortality. While initially beneficial by normalizing wall stress, this hypertrophy may progress to heart failure through unidentified means (4). Although early studies showed that transgenic overexpression of STAT3 in mouse hearts induced pathological cardiac hypertrophy (5), the role of this transcription factor in hypertension-induced cardiac remodeling is still unsettled.

The original suspicion that STAT3 was positively linked to cardiac hypertrophy arose from observations that several members of the IL-6 family of cytokines induced growth of isolated neonatal rat ventricular myocytes. Yet, conflicting findings were reported on consequence of IL-6 deletion on left ventricular hypertrophy and dysfunction resulting from transverse aortic constriction (TAC) (6, 7). Moreover, our lab observed that chronic treatment of mice with the IL-6 family cytokine, leukemia inhibitory factor (LIF) did not induce cardiac hypertrophy (8). More recently, deletion of IL-6 was reported to inhibit cardiac inflammation, fibrosis, and dysfunction in an angiotensin II (AngII) and high salt-induced model of hypertension, without affecting cardiac hypertrophy (9). On the other hand, cardiac-specific overexpression of the STAT3 gene was found to induce hypertrophy of the heart, as well as protection against doxorubicin-induced cardiomyopathy (5).

**Figure 2:**
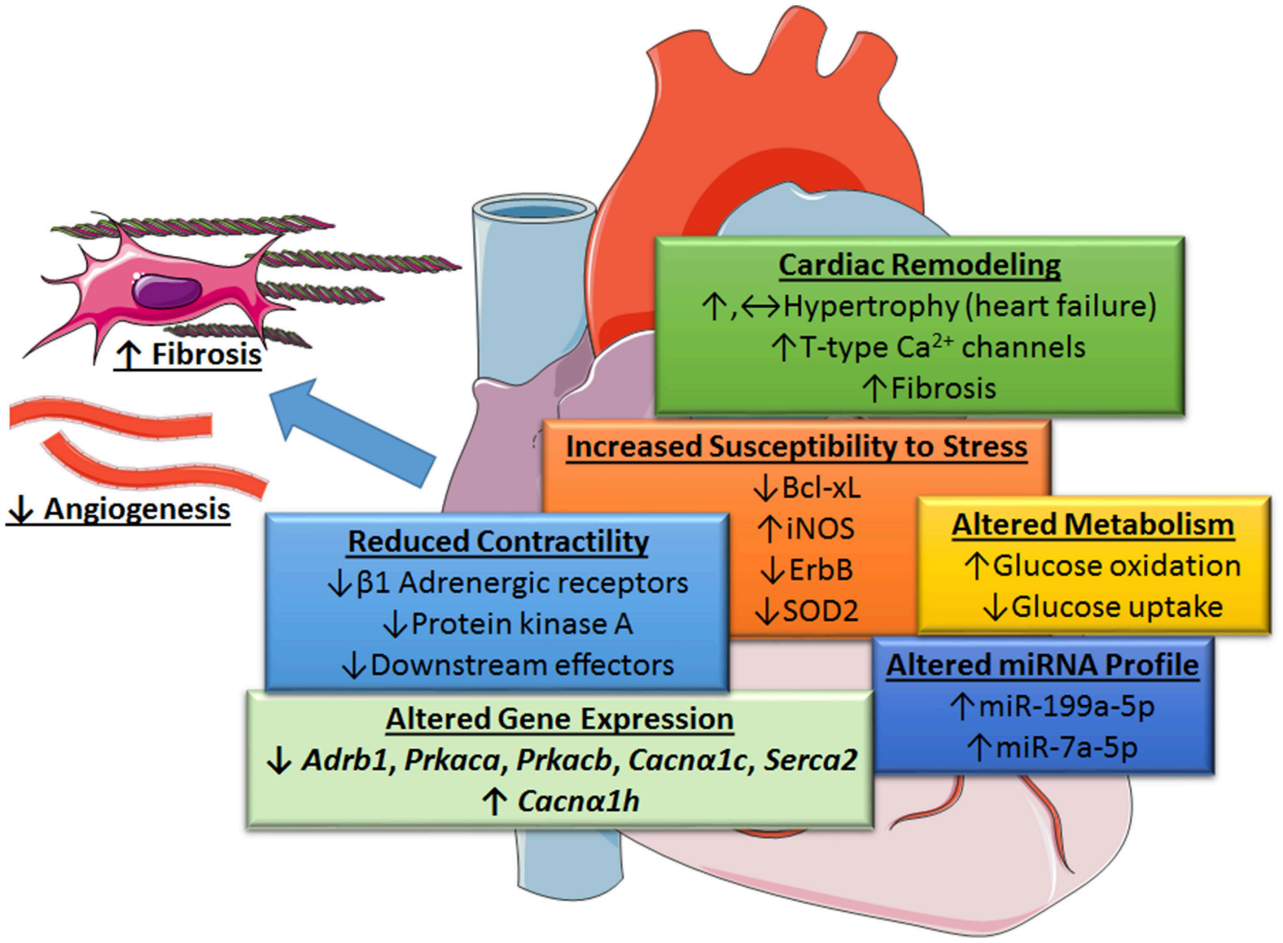
Role of STAT3 in protecting the heart from increased blood pressure revealed by cardiac myocyte-targeted STAT3 deletion. Loss of cardiac STAT3 was found to reduce contractility from β1-adrenergic stimulation and increase susceptibility of the heart to stress by altering gene expression. Under basal conditions, the following genes are downregulated in cardiomyocyte-targeted hearts (↓): β1 adrenergic receptor (*Adrb1*), protein kinase A (PKA) catalytic subunit α and β (*Prkaca* and *Prkacb*), L-type calcium channel subunit (*Cacn*α*1c*), and Serca2; whereas, α-subunit of voltage-gated T-type Ca^2+^ channel (*Cacn*α*1h*) is upregulated (↑), Enhanced levels of certain microRNAs (miRNA-199a-5p and miRNA-7a-5p) induced oxidative stress due to altered glucose metabolism under stress conditions and diminished protective neuregulin-ErbB signaling. Overall, loss of cardiac myocyte STAT3 has a neutral or positive effect (possibly through an increase in T-type Ca^2+^ channels and NFAT activation) on cardiac hypertrophy and associated fibrosis. Evidence suggests, however, that STAT3-deficient cardiac myocytes produce unidentified paracrine factors that attenuate capillary formation and promote fibrosis with aging. Images adapted from Servier Medical Art (https://smart.servier.com/).

Recent gene deletion studies suggest that STAT3 at endogenous levels does not couple to cardiac hypertrophy. Zhang et al. found that cardiomyocyte-restricted STAT3 knockout (KO) mice exhibited marked cardiomyocyte hypertrophy, as well as cell death and associated cardiac fibrosis, in response to chronic β-adrenergic stimulation with isoproterenol (10). The progression of cardiac hypertrophy to heart failure was attributed to an increase in T-type Ca^2+^ channels with subsequent engagement of the hypertrophic transcription factor NFAT (nuclear factor of activated T-cells), as well as the loss of the pro-survival factor Bcl-xl. Acute stimulation was associated with reduced cardiac contractility due to the downregulation of β1-adrenergic receptors, protein kinase A, and several other downstream effectors (Figure 2).

Comparable findings were reported in an AngII-induced model of hypertension (11). In this study, cardiac STAT3 KO mice showed reduced contractile function, but similar hypertrophy as control mice. Moreover, STAT3 deficiency promoted a shift toward increased glucose utilization with cardiac hypertrophy. The results of both of these studies support the idea that STAT3 serves to maintain normal cardiac function in the heart, rather than promoting pathological cardiac growth (Figure 2). However, an earlier study that infused AngII into mice with a global S727A mutation that impedes STAT3 activation showed reduced cardiac hypertrophy and increased reparative patches of fibrosis in the heart (12). The most straightforward explanation for these results is a contribution of non-cardiomyocyte STAT3 to the myocardium under stress conditions.

For a number of reasons the findings arguing against a role for STAT3 in cardiac hypertrophy that are based on its targeted KO in cardiomyocytes may not be definitive, and intriguing reports persists suggesting that STAT3 does play a more active role depending upon the circumstances. Granted many of these studies are also less than definitive as they base their conclusions on an association of STAT3 activity levels with cardiac hypertrophy and/or use a pharmacological inhibitor. Nonetheless, the choice of experimental model may be a determining factor in whether STAT3 contributes to hypertrophy. STAT3 was recently implicated in the mouse heart in ischemia-induced cardiac hypertrophy downstream of heat-shock transcription factor 1 (13). In a mouse model of abdominal aortic constriction, which results in a more gradual increase in pressure overload to the heart and perhaps better mimics the effect of hypertension on the human heart, cardiac hypertrophy was dependent upon poly(ADP-ribose) polymerase 1 (PARP1), in part through its physical interaction with STAT3 and the resultant nuclear accumulation of phosphorylated STAT3 (14). Others have recently reported that endogenous activation of the oxoglutarate receptor 1 (OXGR1) during pressure overload from TAC attenuated cardiac hypertrophy in the mouse by suppressing STAT3 activity (15). OXGR1 is a G protein-coupled receptor that is activated by α-ketoglutaric acid and leukotriene E4. In addition, STAT3 in other cell types besides cardiomyocytes may be important for cardiac hypertrophy. STAT3 in cardiac fibroblasts was linked to the production of paracrine hypertrophic factors, such as ACE and IL-6 (16). Finally, cardiomyocyte targeted STAT3 KO was associated with slowly evolving reduced capillary density after birth (17), indicating other factors may contribute to the phenotype of this model. In this regard, a targeted and inducible knockout model may be more desirable to assess the role of STAT3 in hypertension.

It cannot be discounted that while STAT3 may be pro-hypertrophic it normally inhibits another signaling pathway in cardiac myocytes that couples to hypertrophic growth. Thus, loss of STAT3 and its input into cardiac hypertrophy would be compensated for by activation of this pathway. In this context, the role of STAT3 in regulating mitochondrial function and reactive oxygen species (ROS) formation may be a consideration. In fact, mitochondrial dysfunction is linked to cardiac hypertrophy and heart failure through ROS generation (18). In cardiac myocytes, STAT3 protects mitochondria indirectly via transcriptional means, such as upregulation of the anti-apoptotic protein Bcl2 (19). STAT3 also helps modulate electron complex formation and ROS production via its interaction with the structural subunit of complex 1 GRIM19 or NDUFA13 (20). STAT3 also helps preserve mitochondrial integrity and limit ROS generation via its interaction with cyclophilin D and inhibition of the opening of the mitochondrial permeability transition pore, mPTP (21, 22). Further discussion of the pathophysiological aspects of these extra-genomic actions of STAT3 can be found elsewhere (1, 2).

There is reason to think that endogenous STAT3 may attenuate cardiac hypertrophy in certain cases. An early study showed that IL-10 inhibited isoproterenol- and TAC-induced cardiac hypertrophy (23). Evidence suggested that this was due to inhibition of NF-κB (nuclear factor kappa-light-chain-enhancer of activated B cells) activation, and possibly that of p38, through interaction with STAT3. The observation that left ventricular dysfunction, remodeling (fibrosis and hypertrophy), and fetal gene expression were greater in IL-10 KO mice, supports the physiological relevance of this signaling pathway. A recent study provides evidence for natural killer T (NKT) cells as the source of IL-10 in AngII-induced cardiac remodeling in the mouse (24).

Overall, the findings summarized here indicate that cardiac myocyte-specific STAT3 may not be responsible for hypertrophic growth of the heart in response to a rapid increase in blood pressure. Rather, STAT3 is important for maintaining contractile function and metabolic homeostasis with hypertension. Indeed, endogenous cardiac myocyte STAT3 may attenuate hypertrophy under certain conditions and, in cardiac myofibroblasts, STAT3 likely contributes to extracellular matrix remodeling.

## Cardiac Fibrosis

Besides an increase in the size of individual cardiomyocytes, pathological cardiac remodeling involves increased fibrosis, which is characterized either as reactive or as reparative when replacement of dead myocardium occurs. Multiple lines of evidence indicate that STAT3 is an important contributor to collagen synthesis and cardiac fibrosis (25, 26). New aspects of which are still being discovered and are cited here. With cardiomyocyte-targeted STAT3 deletion, hyper-activated STAT3 was noted in vascular and interstitial myofibroblasts of mouse hearts with chronic β-adrenergic stimulation (10). Relaxin was found to inhibit TGF-β1-induced cardiac fibrosis by blocking STAT3-dependent autophagy in cardiac fibroblasts (27). AngII stimulated STAT3 activation in atrial fibroblasts through an indirect paracrine effect, which was linked to atrial collagen synthesis and fibrosis in the rat (28). In addition, evidence was recently reported that the cell surface transmembrane ligand EphrinB2 in cardiac fibroblasts has pro-fibrotic actions via its synergistic activation of STAT3 and Smad3, and their subsequent association (29).

A complicated interaction involving STAT3 occurs between cardiac myocytes and fibroblasts in regulating fibrosis of the heart under pathological conditions. In cardiomyocytes of mouse hearts subjected to TAC, displacement of STAT3 from the cytoskeletal protein β_IV_-spectrin at intercalated discs, downstream of Ca^2+^/calmodulin-dependent kinase II (CaMKII) activation, was implicated in cardiac fibrosis and loss of cardiac function, but not hypertrophy (30). CaMKII-mediated phosphorylation of β_IV_-spectrin caused displacement of STAT3, resulting in its translocation to the nucleus and induction of profibrotic genes. In another study, cardiac fibrosis in the rat resulting from ligation of the renal artery was attributed to Hsp90-mediated orchestration of IL-6 synthesis by cardiac myocytes, along with its release in exosomal vesicles (31). The resultant biphasic activation of STAT3 in cardiac fibroblasts was implicated in enhanced collagen expression.

## Myocardial Infarction

Cardiomyocyte STAT3 plays an essential role in regulating cardiac remodeling during the subacute phase following an MI (32), although unbridled STAT3 activation is detrimental (33). In part, this involves monocytes/macrophages, which play an essential role in healing the injured heart after MI. Notably, their recruitment to the heart post-MI was found to be facilitated by the secretion of Reg3β by cardiac myocytes in response to the gp130-family cytokine oncostatin M (OSM), and STAT3 was shown to be required for its expression (34). A positive feedback loop sustained by OSM release by infiltrating neutrophils and macrophages was found necessary for proper healing of the infarcted heart.

The initial inflammatory response following an MI is followed sequentially by its suppression and resolution. Proper wound healing is dependent upon reparative macrophages of the M2 phenotype for the repression of inflammation, removal of dead cells and debris, and orchestration of collagen deposition by cardiac fibroblasts (35). Accumulating evidence indicates that STAT3 is a key factor in determining the polarization of macrophages to the M2 phenotype (36). For example, expression of the galectin-3—osteopontin axis by a subset of IL-10-responsive M2 macrophages in the infarcted mouse heart was recently shown to be dependent upon STAT3 (37). Secreted galectin-3 and osteopontin promote the proliferation of fibroblasts and their transformation to myofibroblasts, with collagen synthesis and accumulation, while intracellular galectin-3 promotes osteopontin expression in macrophages. Both proteins also stimulate phagocytosis by macrophages. Others recently reported that in the infarcted rat heart a SGLT2 inhibitor, likely acting as an anti-oxidant, induced the polarization of macrophages to a reparative M2c phenotype that produces IL-10 downstream of enhanced STAT3 activation (38). Fibroblast activation and fibrosis was attenuated by this phenotype by the suppressor cytokine IL-10, as well as by the absence of arginase-1 (an M2a marker) induction, which contributes to collagen deposition and fibrosis.

Unlike the adult heart, the fetal mammalian heart exhibits a regenerative response to MI (39). This response depends upon early resolution of inflammation and expression of the STAT3 target gene VEGFA. The neonatal mouse heart also demonstrates regenerative capacity following the resection of the left ventricular apex. Evidence indicates that cardiac regeneration in this case is a direct transcriptional reversion of the differentiation process that is driven by the Th2 cytokine IL-13, which induces cardiomyocyte cell cycle entry in part via a STAT3-induced periostin pathway (40). This findings is reminiscent of the regenerative program that is initiated by injury in the adult heart of the zebrafish (41). In this model, STAT3 is required for cardiac myocyte proliferation due to the upregulation of the *rln3a* gene, which encodes the hormone Relaxin 3a.

## Peripartum Remodeling

Peripartum cardiomyopathy (PPCM) is a life-threatening condition, which may affect women in the last month of pregnancy or the first months after giving birth. Female mice with cardiac myocyte-specific STAT3 deletion develop a form of PPCM (42). This is associated with blunted superoxide dismutase 2 (SOD2) expression, a gene target of STAT3 (43). The associated increase in oxidative stress leads to increased cathepsin D expression and activity. This in turn forms a pro-apoptotic, anti-angiogenic cleaved form of the nursing hormone prolactin. The 16 kDa shortened form of prolactin leads to increased miR-146a expression in endothelial cells, which exerts angiotoxic effects and upon release in exosomes impairs metabolic activity of cardiomyocytes and reduces their expression of Erbb4, Notch1, and Irak1 (43, 44). Paradoxically, enhanced Akt activity in PPCM, downstream of prolactin or interferon-gamma (IFN-γ), further exacerbates the redox imbalance and loss of SOD2, due to activation of p66SHC and down-regulation of anti-oxidative transcription factor FoxO3A (43). In addition, activation of Akt sustains cardiac inflammation by leading to the induction of the pro-inflammatory chemokine CCL2, which among several actions serves to recruit macrophages. Serum levels of activated cathepsin D and the cleaved prolactin are elevated in PPCM patients (43) and left ventricular STAT3 protein levels are decreased in patients with end-stage heart failure due to PPCM (42).

Low STAT3 ventricular levels were also found to compromise glucose uptake and thereby sensitize the normal and peripartum heart to the toxic effects of chronic β-adrenergic signaling by contributing to a state of energy depletion and associated increased generation of ROS (45). This was accomplished in STAT3 KO hearts by the upregulation of two microRNAs: miRNA-199a-5p, which suppressed glucose transporter-4 (GLUT4) levels, and miRNA-7a-5p, which suppressed expression of the cardioprotective receptor for neuregulin, ErbB. Overall, glucose uptake and oxidation by the heart are reduced upon chronic stimulation with the β-adrenergic agonist isoproterenol. Cardiac myocytes are more reliant on glucose oxidation under these conditions, as isoproterenol depletes serum free fatty acids, and cardiac free fatty acids uptake and triglycerides. Evidence was reported that inadequate glucose uptake by cardiac myocytes from loss of STAT3 was associated with increased mitochondrial ROS formation due to insufficient NADPH generation needed to maintain adequate GSH recycling in mitochondria.

## Myocarditis

Myocarditis or inflammatory cardiomyopathy is most often caused by a viral infection, commonly involving coxsackievirus. Although all ages are at risk, myocarditis commonly affects the young. In most cases, myocarditis resolves spontaneously, but more than 40% of affected individuals progress from increased cardiac hypertrophy, apoptosis, and fibrosis to a dilated cardiomyopathy with reduced contractility (46). Nearly 20% of sudden death among young adults are attributable to myocarditis. Evidence indicates STAT3 couples to potent protective innate immune response in the context of myocarditis. Decreasing IL-6 family cytokine signaling in mice by either cardiac-specific suppressor of cytokine signaling 3 (SOCS3) overexpression or gp-130 knockout was found to increase susceptibility of the heart to coxsackievirus B3 (CVB3) infection, even though an intact IFN-mediated antiviral response was still present. The protective effects of gp-130 signaling was attributed to STAT3-mediated maintenance of dystrophin expression after virus expression, which is important for membrane integrity, as well as STAT3 contributing toward endogenous α-sarcoglycan levels (47). Disruption of the sarcolemmal membrane due to dystrophin cleavage was linked to CVB3-induced death of cardiac myocytes. Mice with cardiac-specific STAT3 depletion exhibited a long-term decrease in cardiac function after virus infection that was associated with cardiac fibrosis due to increased expression of collagen I and reduced matrix degradation (48).

In contrast, genetically prolonged and enhanced STAT3 activity in cardiac myocyte was associated with greater inflammation, left ventricular rupture, and worse outcome following subacute MI (33). Thus, the degree of STAT3 activation in the heart likely has an impact on outcome. In addition, this finding likely illustrates the contribution of spatiotemporal context and concurrent signaling in the outcome elicited by STAT3 activation (49).

**Figure 3:**
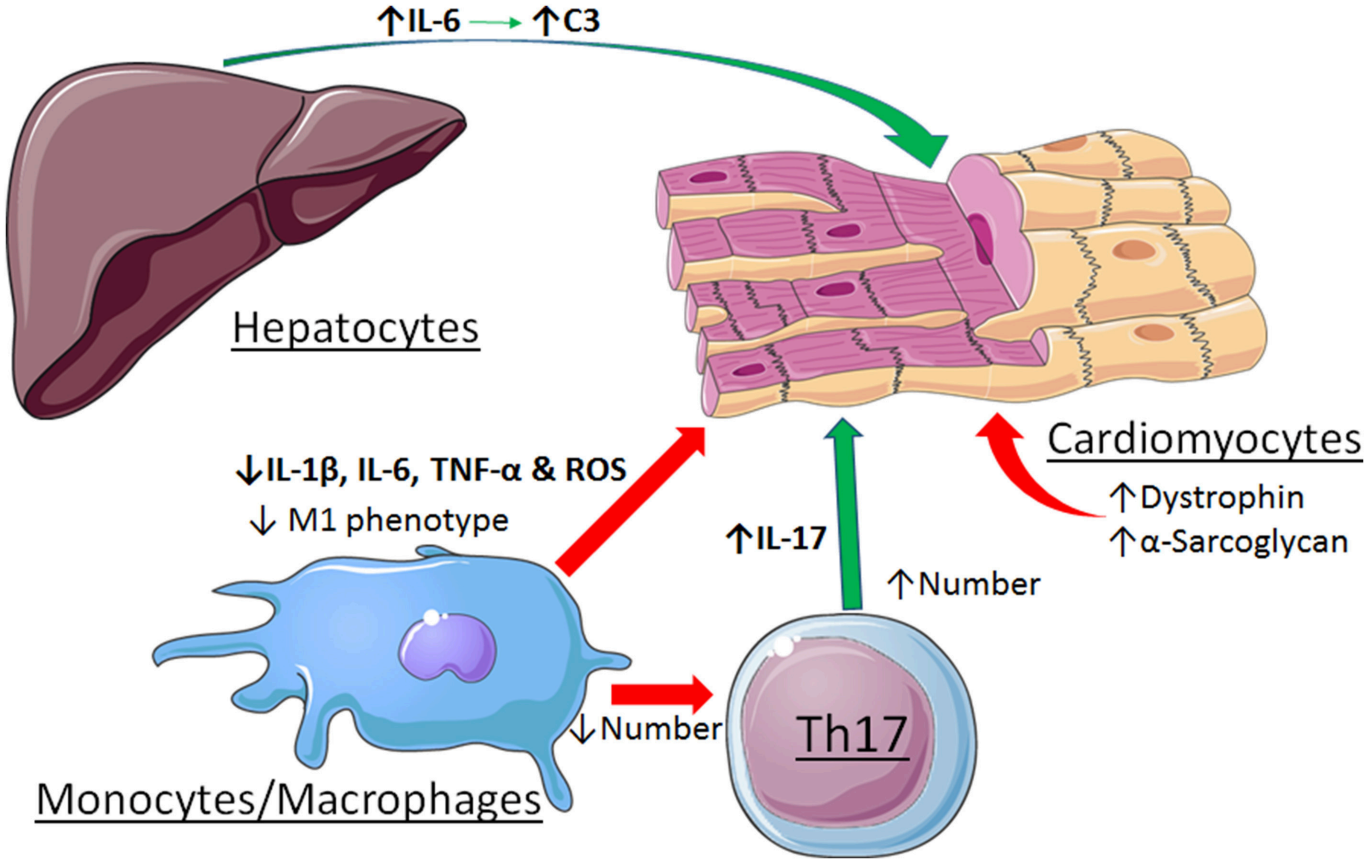
Contrasting roles of STAT3 in viral myocarditis. Cardiac myocyte-specific STAT3 protects against the initiation and development if the dilated phenotype by maintaining expression levels of dystrophin and α-sarcoglycan. Activation of STAT3 by stimulation of the α7nAchR in macrophages alleviates myocarditis by favoring a less inflammatory phenotype. In contrast, STAT3 contributes to myocarditis due to enhanced hepatic and cardiac IL-6 production, as well as IL-6-induced complement component C3 production in the liver. STAT3 also contributes to the differentiation and expansion of Th17 cells that have a role in the development of myocarditis. See text for additional details. Images adapted from Servier Medical Art (https://smart.servier.com/).

Other cell types are involved in the pathogenesis of myocarditis as well, further complicating the role of STAT3. Evidence was reported that STAT3 contributes to immune-mediated myocarditis in mice due to enhanced hepatic and cardiac IL-6 production, as well as IL-6-induced complement component C3 production in the liver (50). STAT3 is also important for the differentiation of Th17 cells, which play a major role in the initiation and development of myocarditis (51). Recently, evidence was reported that strategies to enhance the cholinergic anti-inflammatory pathway in the heart by left stellectomy or treatment with an α7nAchR agonist alleviated viral myocarditis (52, 53). These protective actions have been attributed to activation of Jak2-STAT3 signaling in macrophages and attenuation of inflammatory effects via SOCS3 induction, blockade of NF-κB nuclear translocation via formation of an unphosphorylated STAT3-NF-κB complex, or production of tristetraprolin, an AU-rich element (ARE)-binding protein that destabilizes pro-inflammatory transcripts with AREs in the 3'-untranslated region (54). The disparate roles of STAT3 in myocarditis are summarized in Figure 3.

## Additional Novel Mechanisms

### Nuclear U-STAT3

In the heart, STAT3 uses two mechanisms, canonical and U-STAT3, to regulate the expression of two different pools of genes (1). Canonical signaling entails STAT3 parallel dimers, which form upon Y705 phosphorylation, and tightly bind GAS elements in promoters. Canonical signaling is associated with inflammation (1) and protection from acute ischemic stress by upregulation of cardioprotective and anti-apoptotic proteins, including HO-1, COX-2, Bcl-xL, Mcl-1, c-FLIPS, and c-FLIPL (55–57). Phosphorylation of S727 enhances canonical transcription by increasing recruitment of transcriptional coactivators (2, 58), or attenuates signaling by recruitment of a tyrosine phosphatase (59, 60).

U-STAT3 signaling occurs as a consequence of canonical STAT3 signaling-induced STAT3 expression and is proposed to prolong the inflammatory response initiated by canonical signaling (61). Elevated U-STAT3 levels were found in the nuclei of AngII receptor type 1 (AT1) overexpressing mouse hearts and AngII-treated neonatal rat ventricular myocytes (62). U-STAT3 levels correlated with the degree of hypertrophy, and U-STAT3 was postulated to induce a subset of inflammatory and pro-hypertrophic genes in the heart, including osteopontin and regulator of G protein signaling 2 (62).

Some genes are activated by U-STAT3 via a complex with the unphosphorylated p65 subunit of NF-κB. Binding does not involve GAS elements, but rather a specific DNA element that supports binding of p65 homodimers and cooperativity with U-STAT3 (63). STAT3 S727 phosphorylation is not involved, at least for the interaction of U-STAT3 and p65 subunit (63). The importance of S727 phosphorylation in U-STAT3-induced gene expression is not thoroughly studied, but evidence indicates that it is not important (62, 63).

The mechanism by which U-STAT3 activates target genes not requiring NF-κB is unknown (61), but may involve binding of U-STAT3 to a GAS or GAS-like element. Recently, STAT3 K685 acetylation was found to be required for expression of most U-STAT3-dependent genes, while playing a minor role in the expression of genes by canonical STAT3 signaling (64). It was suggested that K685 acetylation is important for recruitment of p300 or may facilitate STAT3 parallel dimer formation in the absence of Y705 phosphorylation. The possibility that U-STAT3, which tends to form anti-parallel dimers, also acts as a dominant negative mutant protein is a consideration (62).

U-STAT3 also binds to AT-rich DNA sequence sites and sequences implicated in chromatin organization, as well as DNA structures involved in nucleosomal structure and assembly (65). These observations suggest that U-STAT3 may influence chromatin organization. Deletion of Drosophila STAT homolog Stat92E was reported to disrupt heterochromatin integrity and result in transcriptional activation of genes that are not its direct target (66). Stat92E also regulates histone 1 (HI) and histone 3 (H3) function by interacting with heterochromatin protein 1 (HP1) (67, 68). This epigenetic role is disrupted by Stat92E tyrosine phosphorylation and its subsequent translocation to target genes (67). Notably, STAT3 also has a conserved pentapeptide motif (PxVxI) for potential binding of HP1.

### Redox Signaling

Nine of STAT3's 14 highly conserved cysteine residues are redox-sensitive and control its transcriptional activity (1). In many cases, at least *in vitro*, oxidative stress was reported to inhibit canonical STAT3 transcriptional activity. We previously reported that levels of monomeric STAT3 measured under non-reducing conditions were decreased in a redox-sensitive manner in the G_α*q*_ model of heart failure (69), although the pathophysiological relevance of this observation was not established. Recent evidence suggests that the redox-sensitivity of STAT3 may be a regulated process controlling its transcriptional actions. In response to stimulation with IL-6 or oncostatin M, STAT3 associated with the anti-oxidant and protective protein peroxiredoxin-2 in HEK293T cells (70). This resulted in the oxidation of multiple cysteine residues in STAT3's DNA binding, linker, and transactivation domains, with higher order complex formation and attenuated gene expression. In a model of enhanced endogenous H_2_O_2_ generation in cardiac myocytes due to a deficiency in a support protein of mitochondrial complex I, peroxiredoxin-2 expression was elevated, as was dimerized STAT3 (19). These hearts were more resistant to ischemia-reperfusion injury because of STAT3-mediated induction of protective proteins, including Bcl2. Given the importance of oxidative stress in the pathogenesis of cardiac hypertrophy and heart failure, it seems likely that STAT3 redox signaling has functional ramifications under these conditions.

### Tissue-Signaling Cytokines

Recent interest has been focused on cytokines of the innate and adaptive immune systems that mostly target tissue cells, with little if any action on immune cells. Two such cytokines are of particular interest in pathological cardiac remodeling, IL-22 and IL-17. Both are produced by different types of innate/adaptive leukocytes, but exert different actions that are influenced by the tissue inflammatory milieu (71, 72). IL-22 is generally considered protective and regenerative, but can synergize with TNF-α, IFN-γ, and IL-17 under pro-inflammatory conditions. It binds to a heterodimer of the IL-10Rβ-chain and IL-22R, thereby inducing the phosphorylation of the associated tyrosine JAK kinases and activating STAT3, as well as STAT1 or STAT5 depending upon the cell type. IL-22 induces the principal MAPKs as well. Serum IL-22 levels were recently shown to be positively correlated with blood pressure, and in AngII-induced hypertensive mice, evidence was found that IL-22 contributes to systemic and local inflammation, endothelial dysfunction, and increased blood pressure (73). The STAT3 pathway was shown to mediate the effect of IL-22 on endothelial function and blood pressure. Another study showed increased IL-22 and IL-22R1 levels in the hearts of AngII-infused mice (74). Treatment with an IL-22 neutralizing antibody attenuated cardiac hypertrophy, fibrosis, and contractile dysfunction, although a reduction in blood pressure may have contributed to these actions.

IL-17 (in particular IL-17A and IL-17F) is pro-inflammatory and implicated in adverse remodeling of the heart associated with hypertension and viral myocarditis (75, 76). It was recently shown to contribute to inflammatory dilated cardiomyopathy, a major cause of heart failure in persons under 40 years of age (77). This was attributed to the stimulation of cardiac fibroblasts to produce chemokines and cytokines that recruit monocytes/macrophages and polarize them toward a pro-inflammatory phenotype. IL-17 signals through a receptor complex linked via TNF receptor associated factor 6 (TRAF6) and receptor-interacting protein kinase (RIP) to the activation of MAPKs, AP-1, and NF-κB. IL-17A was recently reported to cause apoptosis of cardiomyocytes *in vitro* by inducing expression of inducible nitric oxide synthase (iNOS); however, its simultaneous activation of STAT3 and STAT3's binding to the promoter region damped iNOS gene induction (78). The pathophysiological relevance of this inhibitory mechanism was shown *in vivo* using a mouse myocardial ischemia-reperfusion injury model, where inhibition of STAT3 caused increased iNOS expression and worsened cardiomyocyte apoptosis.

## Conclusion and Perspectives

Recent evidence indicates that endogenous levels of STAT3 in the heart are needed to maintain normal structure, contractile function, and metabolism under stress conditions. This conclusion has importance for hypertension, peripartum cardiac remodeling, and viral myocarditis. A growing theme is that STAT3 may also have benefit in the heart by repressing certain genes, and a better understanding of how that is accomplished is needed. The importance of STAT3 in immune cells and in crosstalk among the various cell types of the heart ought to be resolved, as well as how the various cellular subcompartments of STAT3 are integrated in controlling the genomic and non-genomic actions of STAT3 in response to stress or injury. Network analysis of STAT3 as a sentinel at intercalated discs, mitochondria, sarcoplasmic reticulum, and nuclei under stress conditions ought to be performed. In addition, novel signaling aspects of STAT3, such as U-STAT3, its redox-sensitivity, and the importance of STAT3 in chromatin organization have been described, but their implications for cardiac function and response to stress are not fully understood. Lastly, the role of STAT3 in the heart in response to tissue-specific cytokines is an area that requires further investigation. Understanding the novel and protective aspects of STAT3 in the myocardium could lead to new therapeutic approaches to treat heart disease.

## Author Contributions

MK, CZ, and GB helped write the manuscript and create the figures. GB edited the text.

### Conflict of Interest Statement

The authors declare that the research was conducted in the absence of any commercial or financial relationships that could be construed as a potential conflict of interest.
